# CHIPped balance of proteostasis and longevity

**DOI:** 10.18632/oncotarget.22101

**Published:** 2017-10-27

**Authors:** Wojciech Pokrzywa, Thorsten Hoppe

**Affiliations:** Thorsten Hoppe: Institute for Genetics and Cologne Excellence Cluster on Cellular Stress Responses in Aging-Associated Diseases, University of Cologne, Cologne, Germany

**Keywords:** proteostasis, ubiquitin, E3 ligase, chaperone, aging

Aging is modulated by environmental and physiological changes, involving genetic pathways that are conserved from yeast cells to mammalian organisms. Notably, Insulin/IGF-like signaling (IIS) contributes to the integrity of the cellular proteome and thus defines the aging process and the onset of age-related diseases. The insulin receptor named DAF-2 in *C. elegans* or INSR in mammals regulates fidelity of different transcription factors including FOXO/DAF-16, HSF1/HSF-1, and Nrf/SKN-1, which promote stress response pathways and longevity [1]. Given that increased stress tolerance and protein homeostasis (proteostasis) correlate with longevity, it is however surprising that proteasomal degradation is not affected in aging [2]. Recent observations thus question the concept that an age-related decline of protein quality control pathways actually initiates the accumulation of misfolded proteins and ultimately the collapse of proteostasis. To clarify how protein aggregation accelerates the aging process we addressed the mechanistic regulation of quality control networks from an organismal perspective.

Molecular chaperones and proteolytic machineries coordinate folding and removal of damaged proteins to maintain the cellular proteome. The E3 ligase CHIP is central to this crosstalk, promoting disposal of defective proteins via chaperone-assisted proteasomal degradation (CAP) or chaperone-assisted selective autophagy (CASA) [3]. Proteolysis of misfolded polypeptides prevents an age-related pathological accumulation of protein aggregates, however, CHIP-/- mice do not show a general stabilization of substrates previously shown to be ubiquitylated *in cellulo* [4]. Considering that CHIP knockout mice suffer from premature aging rather argues for specific substrates which control the aging process. Our recent study tackled this conundrum and demonstrated a conserved link to IIS regulation by CHIP-mediated turnover of DAF-2/INSR in *C. elegans, D. melanogaster*, and human cells [5]. DAF-2/INSR is stabilized either by mutations of ubiquitylation sites of the receptor or depletion of the CHIP ubiquitin ligase activity. Chaperone binding is not required for recognition and ubiquitylation of DAF-2/INSR, thus distinguishing IIS regulation from chaperone-assisted quality control. In contrast, DAF-2/INSR regulation is highly selective and not even the related insulin-like growth factor receptor 1 is targeted by CHIP.

Given its function in chaperone-assisted ubiquitylation, CHIP is important for stress resistance. Worms or flies lacking CHIP are highly sensitive to proteotoxic conditions and accumulate oxidatively damaged proteins especially late in life [5]. Thus, CHIP provides a prominent role in maintenance of the organismal proteome especially during the course of aging. This model is further supported by the fact that post-developmental depletion of the E3 ligase is already sufficient to reduce longevity. Proteotoxic stress conditions also affect the degradation of DAF-2/INSR, which can be compensated by CHIP overexpression. Mechanistically, altered INSR turnover is linked to an age-related decline in proteostasis and high increase of misfolded proteins, which redirect the E3 ligase towards chaperone-assisted pathways. In contrast to the induction of molecular chaperones, CHIP level remains unchanged even upon stress or during aging. CHIP cytosolic abundance is reduced upon overexpression of aggregation-prone polyglutamine proteins by fostering its recruitment to inclusion bodies. Consequently, CHIP activity but not protein level becomes limited under proteotoxic stress conditions.

**Figure 1:**
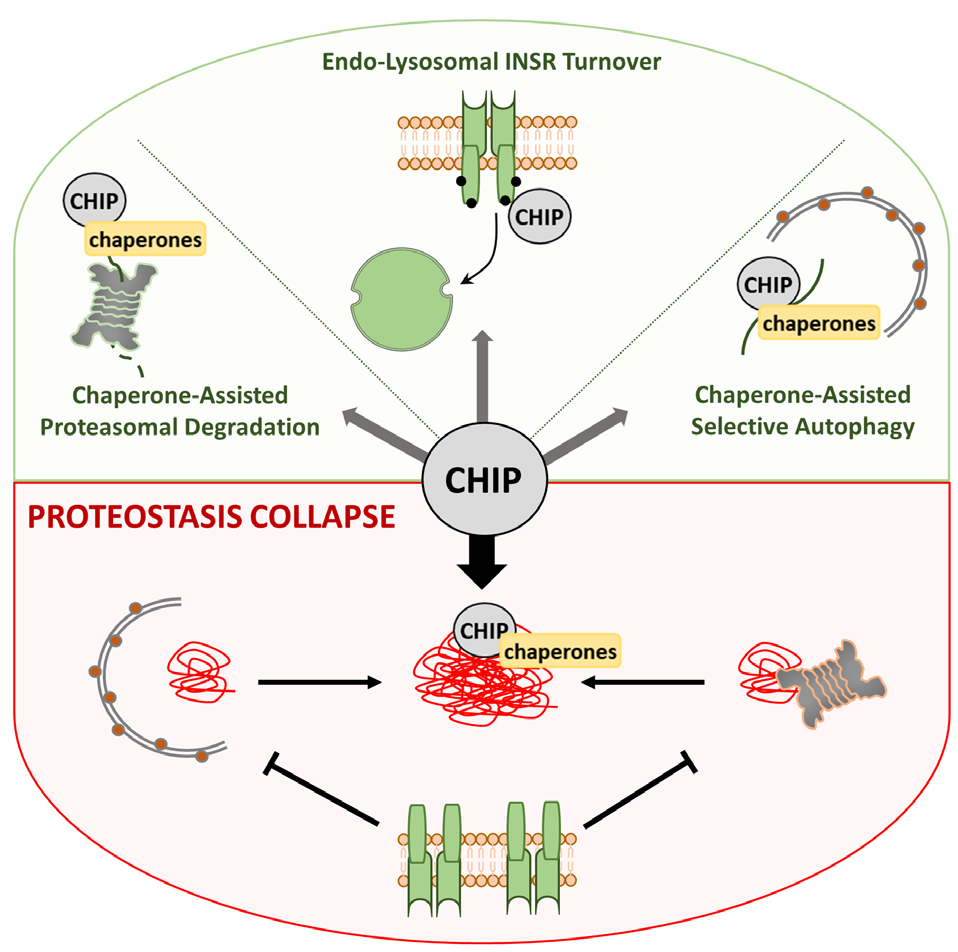
Balanced Selection of CHIP-Pathways CHIP mediates regulation of INSR turnover and chaperone-assisted degradation of misfolded/aggregated proteins by the proteasome or autophagy, which maintains proteostasis and longevity (green). Increased levels of misfolded proteins absorb CHIP activity into protein aggregates, triggering insulin signaling and proteostasis collapse (red).

Thus, the quality control E3 ligase CHIP provides an intricate balance between proteostasis and longevity. Under normal growth conditions, CHIP ensures a healthy proteome by regulating the amount of misfolded proteins and DAF-2/INSR (Figure 1). However, stress or aging-induced rewiring of CHIP degradation pathways triggers a vicious circle that ultimately results in proteostasis collapse and reduced lifespan. Increased DAF-2/INSR level and IIS hyperactivation alleviate the transcriptional induction of stress response pathways, which further boosts the process of protein aggregation (Figure 1). Whereas overexpression of CHIP affects INSR protein level and compensates its stress-induced stabilization in human cells, it does not enhance lifespan of worms and is even toxic in flies. To understand this discrepancy, it is important to emphasize that CHIP teams up with the general Hsp70 and Hsp90 chaperone machinery for recognition of misfolded substrates. CHIP protein levels are obviously highly restricted to avoid unnecessary rerouting of Hsp70 and Hsp90 clients towards degradation [6]. Otherwise, a non-physiological elevation of CHIP would cause a profound imbalance of chaperone and proteostasis networks, thus explaining toxic consequences related to overexpression strategies.

Since CHIP has been considered to function as tumor suppressor, our recent findings are of relevance with regard to cancer development [7]. CHIP regulates the level of numerous oncoproteins such as c-Myc and hypoxia-inducible factor 1α (HIF-1α). Moreover, CHIP is downregulated in different cancer types, inhibiting apoptosis and promoting cell proliferation [7]. Accordingly, drug-based stimulation of CHIP activity appears to be a valuable approach to diminish carcinogenesis, though, deregulation of insulin signaling needs to be counterbalanced. Deciphering the spatiotemporal control of CHIP function as well as mechanisms underlying the regulation of substrate selection and ubiquitylation activity will therefore be essential to develop novel therapeutic strategies.
